# Mast Cells as a Component of Spermatogonial Stem Cells’ Microenvironment

**DOI:** 10.3390/ijms252313177

**Published:** 2024-12-07

**Authors:** Ali Sadek, Yulia Khramtsova, Boris Yushkov

**Affiliations:** 1 Department of Biology and Fundamental Medicine, Ural Federal University Named After the First President of Russia B. N. Yeltsin, 620002 Ekaterinburg, Russia; ali.sadek@urfu.ru; 2 Central Experimental Laboratory of Biotechnology, Institute of Medical Cell Technologies of the Sverdlovsk Region, 620026 Ekaterinburg, Russia; 3 Laboratory of Immunophysiology and Immunopharmacology, Institute of Immunology and Physiology of the Ural Branch of the Russian Academy of Sciences, 620049 Ekaterinburg, Russia; y.hramtsova@iip.uran.ru

**Keywords:** mast cells, niche, microenvironment, spermatogonial stem cells, spermatogenesis

## Abstract

The formation of mature spermatozoa originates from spermatogonial stem cells (SSCs) located near the basement membrane of the seminiferous tubules. This developmental process, known as spermatogenesis, is tightly regulated to ensure continuous sperm production. A critical aspect of this regulation is the balance between SSC differentiation and self-renewal, which is directed by various factors guiding SSCs in either of these two directions. The SSC niche, defined functionally rather than anatomically, includes all factors necessary for SSC maintenance. These factors are produced by cells surrounding the SSC niche, collectively creating the microenvironment of the seminiferous tubules. Coordination between the cells in this microenvironment is essential for the proper function of the SSC niche and successful spermatogenesis. Testicular mast cells (MCs) significantly influence the regulation of this niche, as they contain various biologically active substances that regulate a wide range of physiological processes and contribute to different pathological conditions affecting fertility. This review explores the effects of testicular MCs on SSCs, their role in regulating spermatogenesis under normal and pathological conditions, and their interactions with other components of the testicular microenvironment, with a focus on their potentially critical impact on spermatogenesis and male fertility.

## 1. Introduction

Idiopathic male infertility accounts for about 10% to 20% of all cases [1], indicating a significant knowledge gap in our understanding of the male reproductive system, spermatogenesis, and the associated physiological and pathological mechanisms. This limited understanding, combined with the escalating global infertility rates, has driven increased scientific interest in male reproductive health, as evidenced by the growing number of research publications in andrology, urology, and reproductive medicine [2]. However, not all components of the male reproductive system have received the same level of attention. A quick search of PubMed using the advanced search with the terms “mast cell” and “testis” reveals that only 144 papers have been published from 2000 to the present, which is relatively few compared to other testicular immune cells during the same period, including lymphocytes (1553 papers), macrophages (828 papers), and dendritic cells (348 papers). Moreover, the considerable number of publications on mast cells (MCs) overall (32,473 papers since 2000), with a steady increase over time, emphasizes the relative underrepresentation of testicular MCs in the scientific literature. Meanwhile, MCs are an essential component of the testicular microenvironment surrounding the spermatogonial stem cell niche, playing a crucial role in its optimal functioning. This review explores the influence of testicular mast cells (MCs) on spermatogonial stem cells (SSCs) and their niche, examines the mechanisms underlying their interactions with other components of the testicular microenvironment, and highlights the significance of these interactions in regulating spermatogenesis under both normal and pathological conditions.

## 2. Spermatogonial Stem Cells (SSCs): Between Self-Renewal and Differentiation

The development of stem cell concept (theory) has prompted researchers to reconsider the pathogenesis of many diseases, including those related to impaired fertility. Understanding SSCs and the process of their differentiation and development into fully functional spermatozoa is fundamental to understanding the functioning of the male reproductive system. Located in the basal compartment of the seminiferous epithelium, SSCs play a critical role in maintaining the ability to form spermatozoa throughout a man’s adult life, maintaining the balance between self-renewal and differentiation. The niche serving as the immediate location of SSCs plays an important role in their maintenance. In addition, the microenvironment of the testis, which includes all other cells and compartments surrounding this niche, is important for its optimal functioning.

As established, spermatozoa are produced from a population of undifferentiated cells located on the basal lamina of the seminiferous tubules, termed spermatogonia. These cells were classified into solitary spermatogonia (A_single_ or A_s_), pairs of spermatogonia (A_paired_ or A_pr_), and chains of four, eight, or sixteen cells (A_aligned_ or A_al_). The ratio of A_s_ spermatogonia, which were identified as SSCs, to non-stem cell spermatogonia constitute a mere 0.03% of the total cell population in the adult testis [3]. In the process of spermatogenesis, A_s_ spermatogonia undergo self-renewing divisions, resulting in the generation of two spatially separated A_s_ spermatogonia. This segregation ensures the perpetuation of the stem cell pool. Conversely, when SSCs engage in differentiative divisions, the resultant daughter cells remain physically connected via an intercellular bridge, thereby forming a pair known as A_pr_ spermatogonia. Subsequent divisions of these A_pr_ spermatogonia produce groups of 4, 8, and eventually 16 interconnected spermatogonia, referred to as A_al_. These A_al_ spermatogonia possess the capacity to initiate differentiation into A_1_ spermatogonia, marking the commencement of a series of six generational transitions which are called the differentiated spermatogonia. These transitions proceed through sequential divisions from A_1_ to A_2_, A_2_ to A_3_, A_3_ to A_4_, and finally, from Intermediate (In) to B spermatogonia. The B spermatogonia subsequently divides to form spermatocytes, which, after completing the S phase, advance into the meiotic prophase. Recently, it was found that the majority of A_s_ spermatogonia are predisposed to differentiation, with only a minority serving as true stem cells that are also capable of self-renewal. It has been proposed that A_al_ spermatogonia possess the capability to revert to A_s_ spermatogonia through a process of fragmentation, thereby contributing to the maintenance of SSCs populations. Nevertheless, this proposition is met with skepticism, as several studies present evidence that challenges this notion [4].

The balance between self-renewal and differentiation of SSCs is critically regulated to prevent the depletion of the SSC pool while ensuring an adequate production of differentiated spermatogonia, which ultimately develop into mature spermatozoa by the end of spermatogenesis. However, it is evident that most SSCs are primed for differentiation and display only latent self-renewal capacity [5], which is what explains the very small quantity of SSCs that have the ultimate characteristics of a typical stem cell. Thus, it is important for SSCs to have a special microenvironment which accurately induces and keeps the SSCs in the self-renewal phase to maintain their number.

## 3. The Concept of SSC Niche

Much of our understanding of the physiology and pathology of stem cell microenvironments is based on data derived from studies of hematopoietic tissue. In particular, Friedenstein AJ’s research on the mechanisms regulating the proliferation and differentiation of hematopoietic stem cells through heterotopic bone marrow transplantation under the kidney capsule and skin of mice [6] laid the foundation for investigating the role of the microenvironment in the development of progenitor cells.

The desire of researchers to understand and structure the microenvironment led to the emergence of the concept of a stem cell’s “niche” in 1978, formulated in two works—Schofield R. [7] and Yushkov B.G. et al. [8]. According to Schofield R., the “niche” is a morphological structure in which a stem cell remains in a resting state (G_0_). When the stem cell exits its “niche”—whether through destruction of the niche, migration, or injection into tissue—it begins to proliferate and differentiate. In contrast, Yushkov B.G. et al. describe the “niche” as a temporary functional formation that determines the proliferative activity and direction of differentiation of the stem cell. Thus, the authors introduce the concept of “migratory niche”.

Since then, various stem cell niches have been identified in mammals over the past three decades. These include the hematopoietic stem cell niche found in the bone marrow; niche supporting epithelial stem cells in the skin; niches for intestinal and nerve stem cells; niches of dental pulp stem cells [9]; and the niche for stem cells of the reproductive system [10]. As summarized by Zakhidov S. et al., the works of Clermont Y. et al. established that SSCs are located within the seminiferous tubules in the basal compartment (BC) and are in contact with the basement membrane (BM) [11]. Sertoli cells (SCs) are the only somatic cells directly associated with them [12].

While undifferentiated spermatogonia (A_undiff_) are found along the basal lamina of seminiferous tubules, experiments have shown that SSCs placement is not random. Instead, they tend to congregate in parts of the tubule that are adjacent to areas of testicular interstitial space [13]. This pattern led to the proposal that these specific locations serve as the niche for SSCs. Further refinement of this understanding came from a more recent study, which employed the fluorescent labeling of these spermatogonia and analyzed their distribution in whole mounts of seminiferous tubules. This investigation pinpointed the A_s-pr-al_ spermatogonia’s location more precisely, revealing a preference for regions of the tubule’s basal lamina that are near interstitial vasculature, further defining the SSCs’ niche [14,15]. It has been shown that not only A_s_ spermatogonia, which represent SSCs, occupy this niche but also spermatogonia of other types and preleptotene spermatocytes, which lose contact with the BM. The absence of distinct morphological structures separating SSCs from other spermatogonia in the BC suggests that their niche is not a “restricted niche” containing only stem cells. Instead, it is a “permissive or open niche” that includes stem cells as well as their descendants [3].

This niche structure in the testes likely requires additional regulatory mechanisms or factors to control the behavior of SSCs, beyond those necessary for their function in a restricted niche. There is evidence that these factors can be produced by cells located in the interstitial tissue of the testis [4].

The first data confirming the participation of interstitial tissue in the support of SSCs’ niches in mature mice and rats were obtained by Chiarini-Garcia et al. [16]. Based on data on the morphology of the nuclei of various types of spermatogonia, they demonstrated that SSCs and their undifferentiated spermatogonial descendants of the A_pr_ and A_al_ types are not randomly distributed throughout the BM. Instead, they are specifically located where the tubule contacts the interstitial tissue of the testis. At the same time, differentiated spermatogonia of types A_1–4_, In, and B are located evenly throughout the BM of the seminiferous tubule. Yoshida et al. [14], performing a three-dimensional reconstruction of seminiferous tubule areas containing A_undiff_ and the surrounding interstitial tissue, established that these cells are located on the BM in those areas that border large blood vessels and their branches in the interstitial tissue of the testis. These zones in the interstitium are the richest in Leydig cells (LCs), which confirm their role in supporting the SSCs’ niche. Based on the presented data, we can conclude that the SSCs’ niche is the area of the seminiferous tubule, which is bordered on one side by the BM and on the other by the blood–testis barrier (BTB) and surrounded by Sertoli cells (SCs). This niche is adjacent to the interstitial tissue of the testis and is located near large blood vessels and their branches.

Stem cell niches in different tissues have their own characteristics. In the testis, the niche is divided into two distinct zones: one region is proximal to the interstitial vasculature, where SSCs are mainly located, and a second, slightly more distal but adjacent region, contains A_undiff_ that are ready to differentiate [4]. Support for this theory comes from live cell imaging studies, which have shown that glial cell line-derived neurotrophic factor receptor alpha 1 (GFRα1)-positive spermatogonia are dynamic and actively migrate within or between these regions close to the vasculature. In addition, changes in the vascular pattern surrounding the tubules lead to a change in the position of A_undiff_, bringing them closer to the blood vessels [5]. It is likely that this movement is regulated by a chemoattractant mechanism. Recent studies suggest that the glial cell line-derived neurotrophic factor (GDNF) acts as a chemoattractant for freshly isolated A_undiff_, potentially directing the chemotactic migration of SSCs to areas with higher concentrations of GDNF, essentially the SSC niche [4].

The SSCs’ niche is an ideal location for their self-renewal, which includes all the necessary factors and mediators of this process. The niche definition therefore goes beyond anatomical boundaries and is more accurately described by the presence of specific molecular components and mediators that regulate stem cell function. An interesting aspect of the SSCs’ niche is its dynamic nature, which undergoes changes throughout the seminiferous epithelial cycle, as detailed by Makela and Hobbs [17].

Given the information presented above, the term “niche” can accurately be used to describe the location of the factors required to maintain SSCs. It is also necessary to introduce the term “microenvironment”, which can be used to describe the surrounding components that support the niche and supply it with these important factors.

## 4. The Concept of the Microenvironment Surrounding the SSC Niche

Stem cell niche is the specific location of SSCs, which includes the factors necessary for their maintenance. These factors are localized within a specific region, delineated by SCs, the BTB, and the BM. However, this niche cannot be precisely defined anatomically or morphologically as it lacks clear boundaries separating SSCs from their differentiated progeny, making it an open or permissive niche [18].

Nevertheless, there are morphological distinctions that differentiate the SSC niche from other components of the testes. Based on this understanding, we have divided the SSC microenvironment into two parts: the niche itself and its surrounding microenvironment. The microenvironment supports the niche with all factors necessary for SSCs self-renewal and also supports the differentiation of spermatogonia committed to differentiation. Self-renewal factors such as glial cell line-derived neurotrophic factor (GDNF), colony-stimulating factor 1 (CSF1), vascular endothelial growth factor A 164 (VEGFA164), fibroblast growth factors (FGFs), and chemokine (C-X-C motif) ligand 12 (CXCL12, also known as stromal cell-derived factor 1, SDF1) [13,17]. The differentiation factors are as follows: Neuregulin 1 (NRG1), vascular endothelial growth factor A 165b (VEGFA165b), bone morphogenetic protein 4 (BMP4), activin A, and retinoic acid (RA) [13,17].

**Figure 1:**
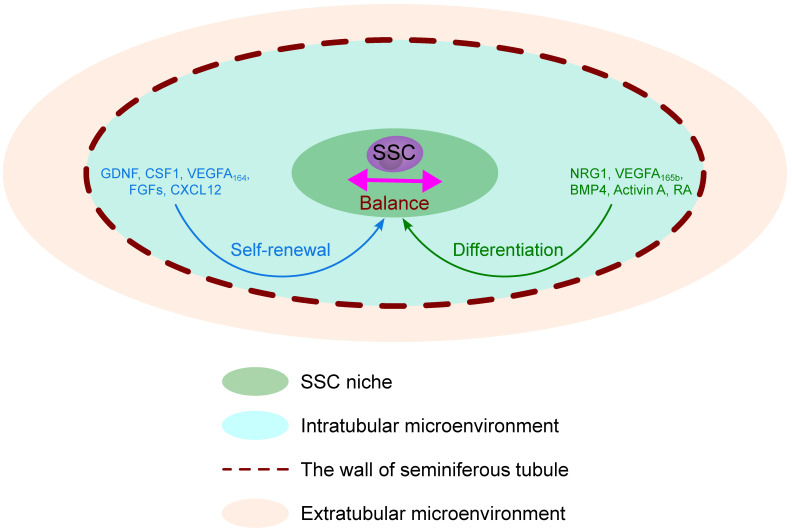
Spermatogonial stem cells’ (SSCs) niche and its surrounding microenvironment. Self-renewal factors: glial cell line-derived neurotrophic factor (GDNF), colony-stimulating factor 1 (CSF1), vascular endothelial growth factor A 164 (VEGFA_164_), fibroblast growth factors (FGFs), chemokine (C-X-C motif) ligand 12 (CXCL12, also known as stromal cell-derived factor 1, SDF1). Differentiation factors: Neuregulin 1 (NRG1), vascular endothelial growth factor A 165b (VEGFA_165b_), bone morphogenetic protein 4 (BMP4), activin A, and retinoic acid (RA).

The microenvironment can be further divided into three main components (Figure 1):

1. The intratubular microenvironment, which includes Sertoli cells (SCs);
2. The wall of the seminiferous tubule (lamina propria), which consists of three layers of peritubular tissue: (1) the inner layer (the basal layer or the basement membrane (BM)), a thick lamella that mainly consists of collagen, (2) the middle layer (the myoid layer) composed of peritubular myoid cells (PMCs) distributed adjacent to the connective tissue lamellae, and (3) the outer adventitial layer (the fibrous layer) com-posed of fibrocytes that originates mainly from the connective tissue of the interstitium [19];
3. The extratubular microenvironment (interstitium), which includes Leydig cells (LCs), immune cells, endothelial cells of blood vessels (TECs), and lymphatic vessels (LECs).

Among the key cellular components of the extratubular microenvironment are MCs.

## 5. Mast Cells as a Component of the Extratubular Microenvironment of the SSCs’ Niche

MCs are of great interest to researchers. Due to the proximity of their location to SSCs and their ability to remodel surrounding tissues, the direct influence of MCs on SSCs is quite possible. Moreover, MCs can play an important role both in maintaining normal spermatogenesis and in promoting the development of pathological conditions when its regulation is disrupted.

MCs are classified into two main subtypes: connective tissue MCs and mucosal MCs. Another classification categorizes them based on their proteolytic enzyme content: MCs containing both tryptase and chymase (MC_TC_) and those containing only tryptase (MC_T_). All these MC subtypes are present in the testes [20].

Testicular MCs in humans can also be classified by location into two types: interstitial MCs and peritubular MCs [21]. Interstitial MCs (globoid MCs) are located near blood vessels or between adjacent Leydig cells and other interstitial cells, whereas peritubular MCs (elongated or tufted MCs) are found near seminiferous tubules or within the lamina propria. Unlike humans, MCs in rats are predominantly localized in the connective tissue of the tunica albuginea (TA) and near subcapsular blood vessels [20].

MCs are present in human testes from the embryonic period, and their number increases in infancy. A subsequent decrease is observed during childhood, followed by an increase at the onset of puberty, indicating a dynamic modulation of MC populations in response to developmental stages [20]. Additionally, MCs are present in other reproductive organs and can be detected using standard histochemical methods, such as toluidine blue, azure, Bismarck brown Y, alcian blue, and safranin staining. They can also be identified through immunohistochemical techniques using markers for MC proteases, including carboxypeptidase, chymase, and tryptase.

**Figure 2:**
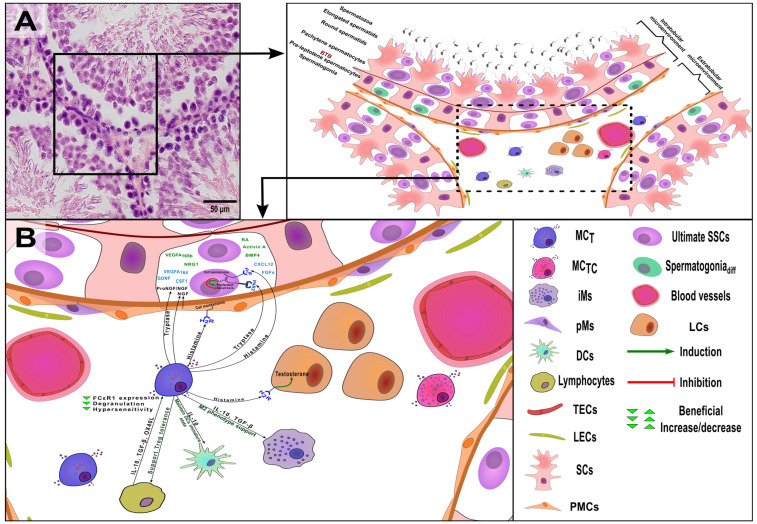
Testicular microenvironment and mast cells (MCs) contributions to their regulation under normal physiological conditions. (**A**) A cross-sectional histological preparation of normal Wistar rat testis (4 months), 400×, H&E; (**B**) Schematic representation of the cross-section of the testis showing the various components of the testicular microenvironment, including their interactions with MCs. The components of the microenvironment secrete various factors for both self-renewal of SSCs (blue) and their differentiation (green). MC_T_—mast cells containing only tryptase, MC_TC_—mast cells containing tryptase, and chymase, iMs—interstitial macrophages, pMs—peritubular macrophages, DCs—dendritic cells, TECs—testicular endothelial cells, LECs—lymphatic endothelial cells, SCs—Sertoli cells, Ultimate SSCs—ultimate spermatogonial stem cells, Spermatogonia_diff_—differentiated spermatogonia, PMCs—peritubular myoid cells, LCs—Leydig cells.

Under physiological conditions (Figure 2), MCs significantly contribute to the immune privilege of the testes and the maintenance of testicular microenvironment homeostasis [20]. This role is largely explained by the regulatory effect of their mediators on vascular permeability and immunomodulation. However, these beneficial effects may shift toward harmful effects when MCs functions are impaired. Such dysregulation can disrupt the balance of the entire testicular microenvironment, negatively affecting the SSCs’ niche itself.

An increase in the total number of MCs, particularly peritubular MCs, has been observed in men with various fertility issues [21]. Additionally, there is a shift from MC_T_-predominant MCs subtype in healthy men to MC_TC_ subtype in patients with fertility problems. The increase in the number of MCs in various fertility disorders suggests their potential involvement in the associated pathologies. This connection has been confirmed by recent studies, which revealed a negative correlation between the number of testicular MCs and certain parameters of the spermatogenic epithelium in cases of non-obstructive azoospermia (NOA) [22]. Moreover, Atiakshin et al. reported a 40% increase in the number of carboxypeptidase A3^+^ (CPA3^+^) MCs in patients with NOA compared to those with obstructive azoospermia (OA). They also propose that chronic inflammation, sustained by MCs via carboxypeptidase A3 (CPA3), contributes to the suppression of spermatogenesis in NOA patients. This suppression negatively affects the outcomes of testicular biopsies and assisted reproductive technologies, resulting in poorer results compared to OA patients [23].

MCs are typically located close to the vasculature, and similarly, SSCs prefer positions near blood vessels within their niche. This proximity facilitates interactions between MCs and SSCs, mediated by the presence of proteinase-activated receptor-2 (PAR-2) on SSCs. Tryptase, a mediator released by MCs, activates PAR-2, suppressing SSCs apoptosis and stimulating their proliferation through increased intracellular Ca^²^⁺ levels [21]. This interaction suggests that MCs play a role in SSCs maintenance but could also potentially contribute to the development of germ cell cancer under certain conditions. Moreover, elevated levels of tryptase have been noted in the seminal fluid of patients with fertility problems, suggesting several of its sources, including MCs of the testes and epididymis [24]. SSCs express histamine receptors. Although histamine receptor 2 (H_2_R) activation has been found to be critical for the maintenance of SSCs, the outcome of histamine receptor 1 (H_1_R) activation is still unknown [25].

A diverse set of mediators and signaling molecules allows MCs to influence and modulate the functions of immune cells of the testis and critical components that directly interact with SSCs, such as SCs and PMCs [21].

## 6. Interaction of MCs with Various Components of the Microenvironment

### 6.1. Interaction of MCs with Components of the Intratubular Microenvironment

**SCs.** Sertoli cells are the main regulators of the SSCs’ niche, since they are the only cells in direct contact with germ cells. SCs make up 40% of all intratubular cells of the testis [26]. Inside the seminiferous tubules, the SCs are the cornerstone; on the one hand, they support the SSCs’ self-renewal by releasing GDNF, fibroblast growth factor 2 (FGF2), probably CXCL12, and, on the other hand, promote spermatozoa production by releasing retinoic acid (RA), which initiates spermatogonia differentiation [17]. Thanks to tight junctions, SCs form the BTB, which provides structural support for the SSCs’ niche and the protective function of germ cells from the immune response, creating immune privilege in the testis. The BTB divides the seminiferous epithelium into two sections: basal and adluminal. In the basal compartment (BC), SSCs, other types of spermatogonia, and preleptotene spermatocytes are located in their niche, while the remaining germ cells are located in the adluminal compartment (AC).

**Figure 3:**
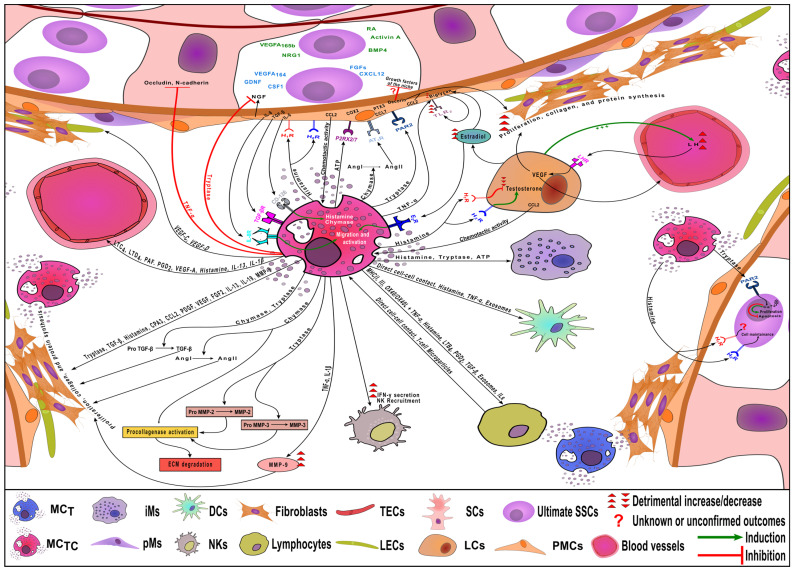
Interactions between testicular MCs with the other components of the SSCs’ microenvironment during spermatogenesis disorders. MC_T_—mast cells containing only tryptase, MC_TC_—mast cells containing tryptase and chymase, iMs—interstitial macrophages, pMs—peritubular macrophages, DCs—dendritic cells, NKs—natural killers, TECs—testicular endothelial cells, LECs—lymphatic endothelial cells, SCs—Sertoli cells, LCs—Leydig cells, Ultimate SSCs—ultimate spermatogonial stem cells, PMCs—peritubular myoid cells.

SCs express various receptors that regulate their function. The direct effect of MCs on the SCs has been poorly studied, but it has been established that tumor necrosis factor-α (TNF-α) can reversibly disrupt the adhesion of the SCs and spermatogenic cells, as well as suppress the expression of occludin and N-cadherin (Figure 3), important components of the tight junctions of the SCs. These data may indicate a direct effect of MCs through TNF-α on SCs and BTB [27].

### 6.2. Interaction of MCs with Components of the Wall of the Seminiferous Tubule

**BM.** The basement membrane of seminiferous tubules consists of two main building blocks: type IV collagen and laminins. The importance of the BM is that it maintains pools of proteases, protease inhibitors, cytokines, and growth factors, many of which are known regulators of SC functions such as BTB integrity and cell cycle regulation. To our knowledge, there is very little research on the role of the BM in regulating the SSCs’ niche and the microenvironment of spermatogenesis, but since the BM consists mainly of collagen IV, this means that it is an ideal target for MCs mediators that activate various matrix metalloproteinases (MMPs) [28]. The effect of MCs on the BM and the impact of this effect on the SCs and the function of the BTB is a rich area for further research.

**PMCs.** Peritubular myoid cells are located around the seminiferous tubules and play a key role in maintaining their structural integrity. These cells act as a bridge between the intratubular and extratubular microenvironments, participating in important signaling processes [29]. PMCs contribute to the support of the SSCs’ niche in variety of ways. They release the niche growth factors GDNF, FGF2, and CSF1, which induce self-renewal of SSCs, as well as the growth factor NRG1, which is necessary for the differentiation of germ cells. In addition, PMCs play an important role in maintaining the function of the BTB by releasing extracellular matrix components such as fibronectin, collagens, proteoglycans (decorin and biglycan), and entactin [29]. Through the release of antioxidant proteins, namely catalase, parkinin/DJ-1, peroxiredoxin 1, superoxide dismutase (SOD) 1 and 2, glutathione-S-transferase and heme oxygenase 1 (HMOX1), PMCs contribute to the protection of SSCs [30]. In addition, they possess receptors for various growth factors, including platelet-derived growth factor and epidermal growth factor, receptors for histamine (H_1_R and H_2_R), TNF-α (TNF_1/2_), and tryptase (PAR2) [29]. They also express androgen receptors, receptor-g, peroxisome proliferator-activated receptor gamma (PPAR-γ), and the intercellular adhesion molecule 1 (ICAM-1).

On the surface of the PMCs, receptors for many MCs mediators are found, which modulate their function, especially in pathology (Figure 3). The exact effect of histamine on PMCs requires further study, but what is currently known is that activation of H_1_R and H_2_R leads to different consequences with evidences of the importance of H_2_R for PMCs maintenance [29]. Tryptase and TNF- α stimulate and enhance PMCs decorin production through the activation of PAR-2 and TNF_1/2_, respectively, which in turn may play a significant role in the development of male infertility [31]. Elevated levels of decorin are observed in the fibrous tubular walls of the testes of patients with infertility, and this is the only difference found between normally functioning PMCs and PMCs with fibrosis [31], which indicates the potential role of MCs in the development of this pathology. Moreover, decorin can interfere with growth factor signaling—further studies are needed to understand its effects on the growth factors of the SSCs’ niche—and plays a role in inflammation [29,32]. Chymase and Adenosine triphosphate (ATP), released from MCs, have the ability to activate the angiotensin receptor AT_1_R (through the generation of angiotensin II) and purinergic receptors (P2X) on PMCs, respectively [29], which leads to the release of pro-inflammatory factors [21]. MCs can induce the expression and secretion of nerve growth factor (NGF) and pro-nerve growth factor (proNGF) by PMCs through their production of TNF-α. The potential roles of NGF and proNGF include their involvement in the second meiotic division of spermatocytes. Their levels may be associated with the sympathetic innervation of the human testis, which could be linked to male infertility. Additionally, NGF and proNGF may also act on MCs by inhibiting their apoptosis [31,33]. In contrast, MCs can release tryptase, which cleaves proNGF and regulates the pro-NGF/NGF ratios [31]. TNF-α is also involved in the overexpression of Toll-like receptor (TLR) on the surface of PMCs, a receptor that is activated by biglycan. This activation increases the production of several inflammatory mediators, including pentraxin-related protein 3 (PTX3), monocyte chemotactic protein 1 (MCP-1), also known as CC chemokine ligand 2 (CCL2), and IL-6. The specific function of PTX3 in the testes, which is mainly secreted by PMCs [34], has not yet been elucidated. CCL2 acts as a chemotactic agent, attracting monocytes and recruiting memory T cells and dendritic cells to sites of inflammation caused by tissue injury or infection. This may explain the significant increase in the number of testicular MCs, particularly in close proximity to the seminiferous tubules (peritubular MCs) and even within the lamina propria during spermatogenic disorders. Furthermore, CCL2 mediates direct profibrogenic effects by signaling fibroblasts to express transforming growth factor-beta (TGF-β), a known collagen production stimulator [35].

PMCs respond to the MCs signal by releasing a variety of mediators that influence the MCs. IL-6 released from PMCs by binding to CD-126 on MCs may lead to increased histamine and chymase production. TGF-β can reversibly inhibit IgE-mediated mast cell inflammatory function through a pathway that diminishes the protein expression of the FcεRI subunit proteins, but not their mRNA expression [36]. IL-8 lead to increased activation and migration of MCs [37].

### 6.3. Interaction of MCs with Components of the Extratubular Microenvironment

**LCs.** Leydig cells release testosterone (T) and estradiol (E). The T/E ratio is an important parameter for spermatogenesis, and its decrease (<10) leads to abnormal sperm parameters [38]. Studies have shown that the proliferation and differentiation of both MCs and LCs occur simultaneously in rat testes, indicating a dynamic interaction between the two cell types mediated by their secretory products [23]. MCs can influence the process of steroidogenesis by acting on LCs histamine receptors. Activation of the H_2_R receptor promotes testosterone production, whereas activation of H_1_R can inhibit it [21,39]. In addition, it was found that the activation and inhibition of testosterone production may depend on the concentration of histamine. Nanomolar concentrations of histamine stimulate testosterone production, while micromolar concentrations prevent it [25,39]. Moreover, at high concentrations of histamine, the inhibition of steroidogenesis was selectively antagonized by an H_1_R antagonist, indicating H_1_R involvement. In contrast, at low concentrations, histamine stimulated steroidogenesis through H_2_R, as this effect was reversed only by an H_2_R antagonist [39,40].

Similarly to PMCs, LCs are a major source of CCL2 in cases of spermatogenic dysfunction, which attracts MCs and leads to their colocalization with LCs. Atiakshin D. et al. reported an increased number of CPA3^+^ MCs in patients with NOA, particularly in proximity to LCs. They suggested that MCs, by localizing near LCs, may attempt to enhance steroidogenesis to boost spermatogenesis through the release of histamine and potentially through the secretion of CPA3. This process, which aims to correct the distorted spermatogenesis, could simultaneously trigger a local inflammatory reaction and create profibrogenic tissue niches due to the excessive secretion of MC mediators [23]. Additionally, LCs, due to their excess production of estradiol, may induce the migration and activation of MCs, as MCs express estradiol receptors (E2R) [21].

**Testicular immune cells.** Mammalian testes contain diverse immune cells within the extratubular microenvironment, including testicular macrophages, dendritic cells (DCs), and T-lymphocytes. In normal spermatogenesis, the number of resident immune cells present in the testicular interstitium is low relative to other somatic cell types [41].

**Macrophages.** like MCs, are innate immune cells that differentiate from early myeloid precursors and are located in all tissues of the body. These are the most abundant testicular immune cells, and in rodents they constitute 20–25% of all interstitial cells. Testicular macrophages can be divided according to their location into two types: peritubular macrophages (pMs) and interstitial macrophages (iMs). pMs do not have a specific shape and are located in areas close to the SSCs’ niche. They express CSF1, which induces spermatogonia proliferation and differentiation, and enzymes involved in the biosynthesis of RA. Temporary depletion of pMs leads to impaired differentiation of spermatogonia [42]. In contrast, iMs are the most common immune subtype in the testes [41]. They have a round, oval or heterogeneous shape depending on the animal species [43] and are located inside the interstitium of the testis in close contact with the LCs. Testicular macrophages can also be divided into two phenotypes based on their functions: M2 (anti-inflammatory/healing phenotype) with surface expression (CD68^−^ CD163^+^), which are considered resident testicular macrophages, and M1 (inflammatory/killing phenotype), which is divided into intermediate macrophage with surface expression (CD68^+^ CD163^−^), and infiltrating macrophage with surface expression (CD68^+^ CD163^+^) [44]. Normally, testicular macrophages exhibit an M2 phenotype and secrete high levels of IL-10 and low levels of TNF-α, which maintain immune homeostasis and a privileged testicular environment [45].

Under normal physiological conditions, testicular MCs exhibit immunosuppressive properties through the production of anti-inflammatory factors such as IL-10 and TGF-β, which maintain the M2 macrophage phenotype. However, after activation, MCs can release ATP, which, by binding to NLR family pyrin domain containing 3 (NLRP3) (previously known as NACHT, LRR, and PYD domains-containing protein 3, NALP3) on macrophages, leads to their switching to the M1 phenotype [46]. MCs, through the release of various mediators, can activate various MMPs that degrade extracellular matrix components. Some of these cleaved components can, in turn, activate macrophages. For example, fragments of fibronectin generated during this process serve as stimuli for macrophages [46]. Tryptase released by MCs activates PAR2, which is expressed by testicular macrophages, inducing their proliferation and cytokine production [47]. Moreover, similar to Leydig cells (LCs), testicular macrophages respond to histamine in a concentration-dependent manner. Khan and Rai reported that at high concentrations, histamine reduces both phagocytosis and superoxide production in testicular macrophages. Conversely, at low concentrations, superoxide production increases while phagocytosis remains unaffected. This difference may arise because the machinery involved in phagocytosis is less sensitive to histamine than that regulating superoxide production [39].

The same study found that histamine’s effects are not only dose-dependent but also receptor-specific. The inhibitory effects observed at high concentrations were counteracted by an H_1_R antagonist, whereas the effects at low concentrations were blocked by an H_2_R antagonist but not by an H_1_R antagonist [39].

It is important to note that studies on histamine’s effects on testicular macrophages are limited, and the work of Khan and Rai was conducted on wall lizards. Additionally, there are significant variations in histamine’s effects depending on the tissue origin of macrophages. For example, Azuma et al. demonstrated that histamine inhibits chemotaxis, phagocytosis, and superoxide production in rat peritoneal macrophages in a dose-dependent manner, with the inhibitory effect mediated by H_2_R rather than H_1_R [48]. In contrast, Triggiani et al. found that histamine stimulates immune responses in human lung macrophages [49]. These discrepancies in results, depending on tissue type and species, highlight the need for further investigation into the effects of histamine on testicular macrophages, preferably in humans or, at the very least, in mammals.

**DCs.** Dendritic cells represent a minor population of interstitial cells in the normal testis. DCs are migratory cells that move between peripheral organs and lymph nodes through lymphatic drainage or blood vessels. Under physiological conditions, testicular DCs exhibit an immature phenotype. Consequently, they do not activate lymphocytes, indicating that they maintain a tolerant status. However, in pathologies, in particular in experimental autoimmune orchitis (EAO), their number increases significantly, which indicates their participation in the autoimmune response. It has been proven that the observed increase in the number of DCs in the testes of rats with EAO is associated with the recruitment of new progenitor cells and DCs differentiation in situ. Both in vitro and in vivo data indicate that MCs can potentially influence DCs migration, maturation, and function [50].

To our knowledge, there is limited data on the interaction between MCs and DCs. However, their close proximity facilitates direct contact between these cells, and the numerous cytokine and chemokine receptors expressed by DCs enable them to respond to various soluble mediators released by MCs. Consequently, MCs and DCs may collaborate in modulating immune responses to alterations in the testicular environment. For instance, in the skin, it has been demonstrated that MCs secrete IL-10, which not only reduces DCs differentiation but also enhances their ability to inhibit T cell proliferation and cytokine production [51]. Direct contact between MCs and DCs may result in upregulation of DCs maturation markers, increased expression of Th2-specific cytokines, increased DCs chemotaxis to the lymph nodes, and increased their ability to stimulate T cells. It has been shown that the supernatant of activated MCs induces activation of DCs maturation markers, increases CCL21 chemotaxis to lymph nodes and stimulate Th2-stimulating DCs [52]. In particular, TNF-α and histamine play a major role in these changes. TNF-α acts locally to increase the expression of E-cadherin, which, in combination with other inflammatory cytokines, serves to attract DCs to the site of infection. Histamine improves endocytosis, antigen cross-presentation, and induces CD86 expression and chemokine production by immature DCs. In addition, histamine has been shown to alter the pattern of cytokines secreted by mature DCs and hence their ability to polarize T cells toward a Th2 response. In addition, histamine increases the ability of DCs to stimulate the proliferation of memory T cell populations [52,53]. The interaction between MCs and DCs can shift the immune response toward the Th2 pathway. However, Dudek et al. found that this interaction can redirect the immune response toward the Th1 and Th17 pathways. The authors explain that previous studies have mainly focused on the modulation of DCs by MCs mediators released during IgE-triggered degranulation. In addition, many of these studies examined the effects of MCs mediators on DCs function in the presence of strong maturation factors such as LPS, which could bias the results toward the Th2 pathway. Another mechanism by which MCs can regulate DCs function is through exosomes. Exosomes derived from MCs have a high ability to induce maturation (expression of CD40 and CD80) and functional activation (secretion of IL-12p70) of DCs and can potentiate the efficiency of antigen presentation by DCs [50].

**Lymphocytes.** Normally, lymphocytes are always found in interstitial space, consisting of about 10–20% of the testicular immune cell population. Different subpopulations have been identified in the testes of humans and various animals. Regulatory T cells CD4^+^ CD25^+^ Foxp3^+^ (Treg) are found mainly in the subalbuginal and peritubular areas [54], effector T cells (CD4^+^ T cells (less commonly in normal testes), CD8^+^ cytotoxic T cells) and natural killer cells (NK) are found in the interstitium of the testis. B lymphocytes are not found in the testes even during inflammation [55,56]. Treg cells play an important role in providing immune privileges by increasing tolerance to sperm antigens. Treg cells of pelvic lymph nodes specifically proliferate in response to testicular antigens and suppress the proliferation of effector T cells.

The close proximity of MCs and lymphocytes at sites of tissue inflammation [50] suggests that MCs and lymphocytes can influence each other’s functions through bidirectional cell–cell interactions. Moreover, MCs can influence T cells indirectly, mainly through their effects on DCs.

**Tregs.** Tregs, through a contact-dependent mechanism, and their production of soluble factors such as TGF-β and IL-10 may lead to a decrease in FcεR1 expression on MCs. In addition, they can suppress degranulation and immediate hypersensitivity via OX40L on MCs [57]. In turn, MCs are important contributors in promoting tolerance through their interactions with Tregs [55]. However, in pathology, it has been found that MCs activation can break Treg tolerance through a mechanism involving a combination of direct cell-to-cell contact dependent on OX40/OX40L interaction and T cell-derived IL-6, resulting in a shift in Treg cells toward Th17 with loss of their immunosuppressive properties [57].

**Effector T cells.** The incubation of MCs with activated T cells causes MCs degranulation and the release of their mediators/proteases. Moreover, their interaction leads to the release of MMP-9. The ability of T cells to activate MCs is achieved through ICAM-1/LFA-1-dependent cell–cell contact and interaction between LTα1β2 and/or LIGHT on activated T cells and LTα1β2 receptors expressed on MCs. In addition, Shefler et al. found that activated T cell microparticles were able to induce the production of soluble mediators from MCs cultures [58].

On the other hand, MCs express MHC class I and II molecules and can process and present antigen in vitro [59]. The interaction between OX40 on activated CD4^+^ T cells and OX40L expressed by MCs, along with the secretion of soluble TNF-α from MCs, co-stimulates the proliferation, activation, and cytokine production of CD4^+^ T cells. MCs can influence T cells activation by releasing exosomes, which induce lymphocytes proliferation and cytokine production in vitro and in vivo. MCs significantly influence T cell activation through various mediators. Histamine, leukotriene B4 (LTB_4_), prostaglandin D2 (PGD_2_), and TNF-α are among the products that facilitate lymphocyte migration, recruitment, maturation, and activation. In particular, PGD_2_ is known to enhance cytokine production by Th2 cells. Histamine has dual contradictory effects on T cells activation: it promotes the activation of Th1 cells through H_1_R and simultaneously suppresses the activation of Th1 and Th2 cells through H_2_R [50]. In addition, MCs are abundant producers of various cytokines, including interleukins such as IL-4, IL-6, IL-10, IL-13, as well as TGF-β. These cytokines play a critical role in directing the polarization of naive T cells into Th1, Th2, Th17, and Tregs, as well as in modulating the functions of various T cell subsets [50].

**NKs.** Activated MCs can induce natural killers’ accumulation in various diseases. For example, immune surveillance by MCs is important for NK recruitment and virus clearance. This NK recruitment occurs through XCL8 and CXCR1, highlighting the role of NKs as sentinel cells during early viral infections. LPS-activated MCs induce cell contact-dependent secretion of IFN-γ by NKs without affecting cell-mediated cytotoxicity. Cellular interactions are mediated in part by OX40L expressed on MCs. Stimulation of MCs by TLR_3_ and TLR_9_, but not IgE/antigen, enhances IFN-γ secretion by NKs. On the other hand, in the tumor microenvironment, CSF-activated MCs release adenosine, which inhibits the production of IFN-γ by NKs [57].

**Testicular Endothelial Cells (TECs), Lymphatic Endothelial Cells (LECs).** Endothelial cells are critical components of the SSCs’ niche, as mentioned previously. TECs, but not the endothelium of other organs, express almost all the growth factors necessary to maintain SSCs in culture and include GDNF, FGF2, stromal cell-derived factor-1 (SDF-1), Macrophage Inflammatory Protein 2 (MIP-2) and insulin-like growth factor binding protein 2 (IGFBP-2). It was found that transplantation of TECs alone into busulfan-treated testes (a model of impaired spermatogenesis) resulted in restoration of spermatogenesis. In addition, injection of TECs into the testes immediately after busulfan treatment protected spermatogenesis [60]. LECs located close to the vasculature express FGF4, 5, and 8, which have been shown to regulate the density of GFRα1-positive undifferentiated spermatogonia (SSCs) [61]. Through the production of FGF, LECs act as key regulators of SSCs population size.

MCs are well known to influence vasculature through various mediators, such as leukotriene C4 (LTC4), leukotriene D4 (LTD4), platelet-activating factor (PAF), prostaglandin D2 (PGD2), vascular endothelial growth factor A (VEGF-A), histamine, IL-13, and IL-1β, which affect TECs [62], as well as vascular endothelial growth factor C (VEGF-C) and vascular endothelial growth factor D (VEGF-D), which influence LECs [63,64]. In cases of male infertility, such as varicocele, impaired testicular circulation is observed, which may indicate the role of testicular MCs in these changes.

## 7. The Role of Mast Cells in Spermatogenesis Disorders

To date, several clinical observations and experimental data have accumulated indicating a possible connection between male infertility and the MCs of the male reproductive organs. However, the exact mechanism behind this connection remains poorly understood. Understanding how MCs are activated and how they respond to various changes in the testicular microenvironment is considered a key step toward clarifying this link. The diverse range of receptors on the surface of MCs, along with the variety of mediators they produce, allows them to receive signals from testicular cells. At the same time, they send signals through these mediators, influencing not only surrounding cells but also themselves. This creates a closed cycle, where MCs receive signals (e.g., recruitment signals from PMCs and LCs via CCL2) and amplify the response by signaling themselves, leading to further recruitment and activation. This closed cycle complicates the investigation of cause-and-effect relationships. Furthermore, although the activation and degranulation of MCs can be triggered by immunoglobulins, lymphocytes, and immunoglobulin-antigen complexes, they can also be activated non-immunologically. The latter can be caused by radiation, pathogens, proteins, proteolytic enzymes, opioids, estrogens, and androgens [65]. Recently, it was discovered that even mechanical pressure and high temperature can lead to activation and induction of mast cell degranulation. This finding suggests that MCs can influence the testicular microenvironment not only through immune responses (e.g., following injury or infection) but also in response to elevated testicular temperature caused by disease or external factors.

### 7.1. Thermal Factor

The testes maintain a temperature lower than the core body temperature [66], as elevated temperatures negatively affect germ cells and disrupt spermatozoa functionality. Studies on MCs reveal that temperature plays a critical role in their behavior. For example, dermal MCs can be activated in vitro when exposed to high temperatures (43 °C) [67]. It has been suggested that MC activation is linked to the expression of Transient receptor potential cation channel subfamily V member 2 (TRPV2) receptors, which respond to mechanical pressure and temperature stimuli [68,69]. Certain pathophysiological causes of male infertility, such as varicocele and cryptorchidism, are associated with increased testicular temperature. Additionally, factors such as lifestyle and behavioral influences, occupational, and environmental factors can also contribute to elevated testicular temperature [70]. This increased temperature may trigger MCs activation and subsequent degranulation within testicular tissue. The degranulation releases various mediators that can significantly alter the microenvironment of the SSCs’ niche, potentially disrupting the balance required for proper germ cell development.

### 7.2. Fibrosis

Testicular fibrosis is one of the most common indicators of male infertility. MCs play an active role in promoting fibrosis in various organs, including the testes. When activated, testicular MCs can directly induce fibrosis by releasing several factors such as tryptase, TGF-β, histamine, CPA3, CCL2, platelet-derived growth factor (PDGF), VEGF, FGF2, IL-13, IL-19, and MMP-9 [20,23]. Moreover, they can induce it and accelerate its development indirectly by affecting other factors. Tryptase converts proMMP-2 and proMMP-3 to their active forms MMP-2 and MMP-3, respectively. MMP-2 and MMP-3, in turn, can activate procollagenase, which promotes tissue damage, remodeling, and fibrosis [28]. While tryptase activates procollagenase indirectly, chymase activates it directly. In addition, chymase, like tryptase, transforms proTGF-β into its activated form. TNF-α and IL-1β lead to the activation of MMP-9, which actively promotes fibrosis. Testicular fibrosis leads to thickening of the walls of the seminiferous tubules, disrupting the necessary signaling processes between its inner and outer microenvironments [71]. MCs may also promote fibrosis by affecting other testicular cells. MCs, by activating PAR-2 on PMCs, induce the expression of decorin which play a significant role in the activation and proliferation of fibroblasts [31]. MCs can negatively affect testosterone levels, depending on histamine concentration and the type of histamine receptor involved [21,25], which theoretically leads to positive feedback from LCs to the pituitary gland, resulting in an increase in luteinizing hormone (LH) levels. Elevated LH levels have been found to induce the expression of VEGF by LCs [72]. VEGF is well known for promoting fibrosis and angiogenesis [73]. However, this hypothesis requires experimental validation.

## 8. The Prospect of MCs in Male Reproduction

Considering the reviewed impact of MCs on SSCs and the testicular microenvironment (Table 1), they present a potential target for therapeutic modulation. Modulation of MCs can serve two purposes. First, it can be used in experiments to more precisely determine their role in regulating the testicular microenvironment and spermatogenesis as a whole, which remains largely unexplored. Second, it can act as a potential approach to addressing certain male fertility disorders. Several studies have investigated the effects of MCs blockers on sperm parameters, reporting positive outcomes [74,75,76,77]. However, modulation is not limited to the inactivation of MCs, it also includes attempts to activate them, investigate the outcomes of their activation, and identify their potential activators. To the best of our knowledge, there is a significant gap in studies exploring this aspect.

## 9. Conclusions

In conclusion, the regulation of the SSC niche by various components of the microenvironment, including MCs, is critical for the formation of functional spermatozoa under physiological conditions and for the restoration of spermatogenesis in various pathologies. Although some studies have investigated the status of testicular MCs in certain types of male infertility, their role in regulating the testicular microenvironment remains poorly understood—particularly regarding their responses to extreme conditions and the mechanisms underlying their interactions with other components of the testicular microenvironment.

It is worth noting that, due to the limited number of studies specifically focused on testicular MCs, we have drawn upon evidence from interactions between MCs and somatic cells (particularly immune cells) in other organs, including reproductive organs, to hypothesize potential interactions between different cellular components of testicular microenvironment. While these hypotheses require validation through experimental studies, the presence of relevant receptors on testicular MCs for substances secreted by various microenvironment components—and vice versa, receptors on microenvironment cells for MCs mediators—provides strong evidence supporting these potential interactions.

Thus, our review highlights the current gaps in knowledge regarding the role of testicular MCs and proposes hypotheses that may serve as a foundation for future research. Addressing these gaps is essential for elucidating the causes of specific pathologies and could potentially lead to new treatment options through the modulation of MC activity.

## Figures and Tables

**Table 1:** Testicular disorders associated with MCs dysfunction.

MC Mediator	Target	Disorder	References
Histamine	LCs	Reduced steroidogenesis	[21,25,39,40]
ATP, chymase, CPA3, exosomes, histamine, ILs, LTB4, PGD2, TGF-β, TNF-α, tryptase	PMCs and immune cells, TECs, LECs	Induction and promotion of pro-inflammatory reactions	[21,46,47]
Tryptase, VEGF family	SSCs, TECs, LECs	Germ cells cancer and angiogenesis	[21,62,63,64]
TNF-α	Occludin and N-cadherin	Disturbances in Sertoli cells tight junctions	[27]
Tryptase, TGF-β, histamine, CPA3, CCL2, PDGF, VEGF, FGF2, IL-13, IL-19, MMP-9, TNF-α	PMCs, fibroblasts, MMPs, and immune cells	Testicular fibrosis and tissue remodeling	[20,23,28,31]
